# Supplementary figures and images for: Suppression of retinal degeneration by two novel ERAD ubiquitin E3 ligases SORDD1/2 in Drosophila
Source: PLoS Genet. 2020 Nov 2;16(11):e1009172. doi: 10.1371/journal.pgen.1009172 (PMC7660902; doi:10.1371/journal.pgen.1009172)

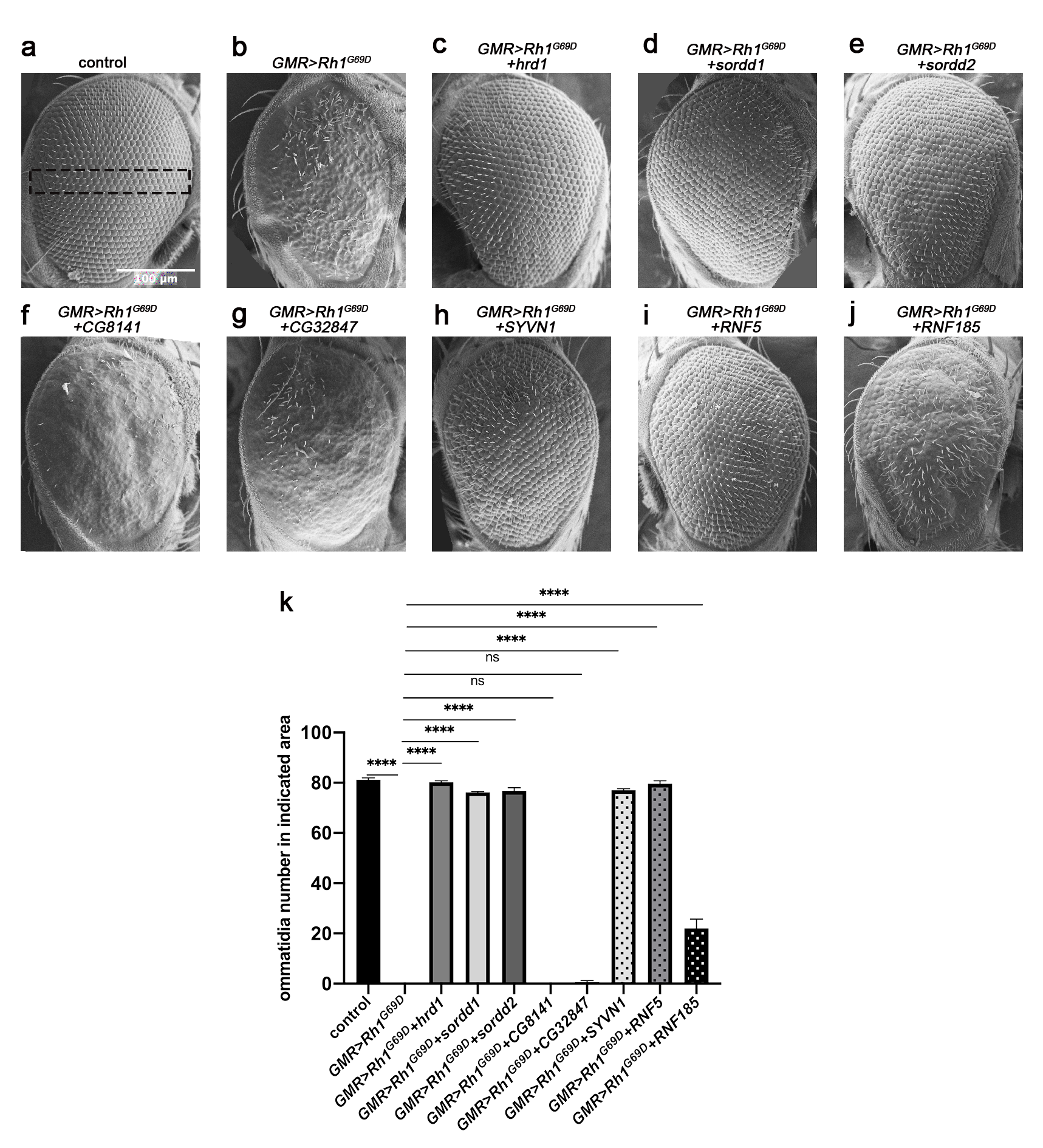

Supplement: S1 Fig — (a-j) Scanning electron microscopy images show eye morphology of (a) control (GMR-gal4/+), (b) GMR>Rh1G69D, (c) GMR>Rh1G69D+hrd1, (d) GMR>Rh1G69D+sordd1, (e) GMR>Rh1G69D+sordd2, (f) GMR>Rh1G69D+CG8141, (g) GMR>Rh1G69D+ CG32847, (h) GMR>Rh1G69D+SYVN1, (i) GMR>Rh1G69D+RNF5 and (j) GMR>Rh1G69D+RNF185. Scale bar, 100 μm. (k) Quantification of the number of ommatidia in the middle 3 rows of a-j (relevant rows are indicated in a). Error bars indicate SEM (n = 5); ns, not significant; ****p<0.0001 (one-way ANOVA, Sidak's multiple comparisons test). (TIF) [file pgen.1009172.s001.tif]

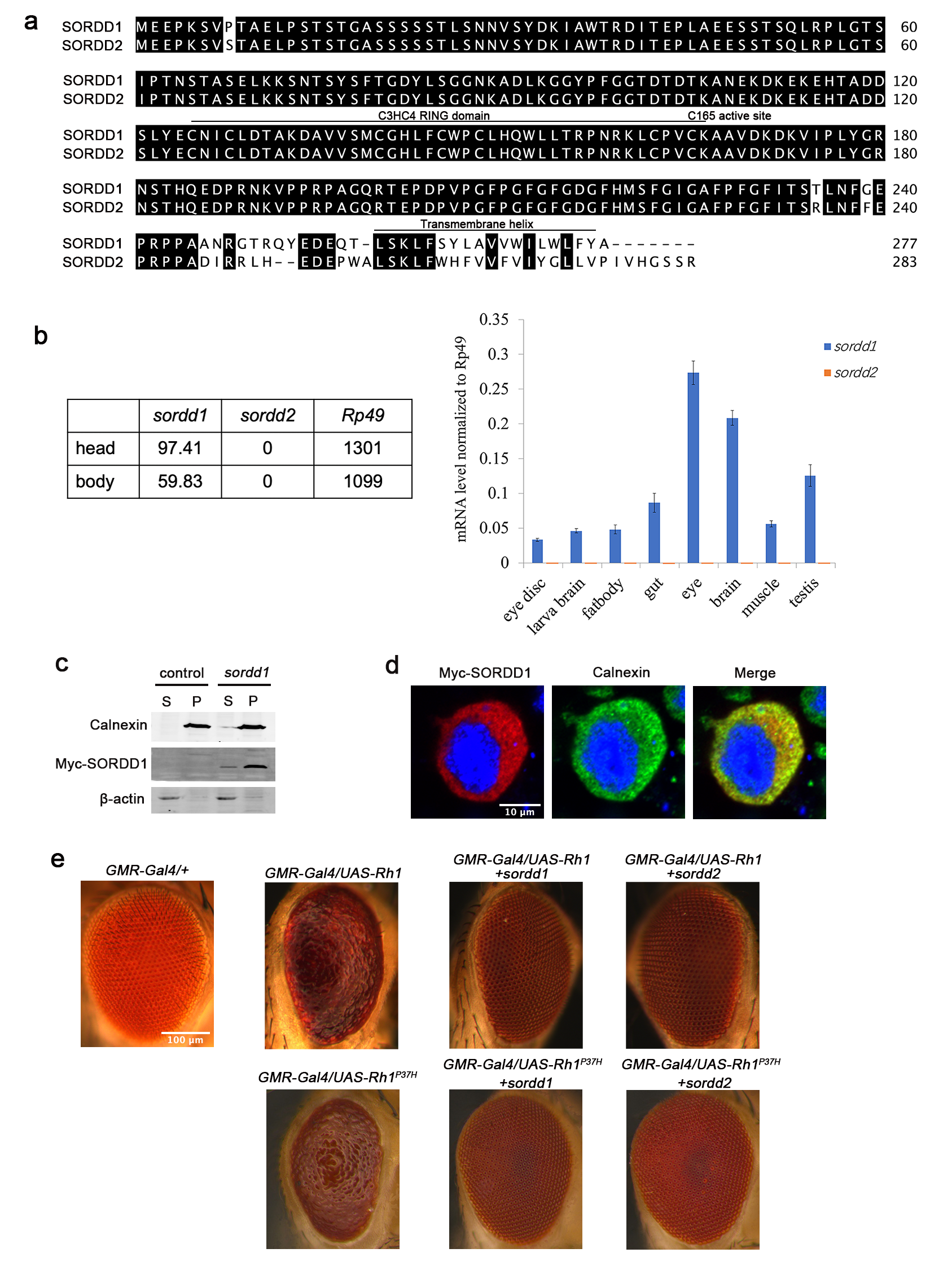

Supplement: S2 Fig — (a) Protein sequence alignment between SORDD1 and SORDD2 showed they share 92.4% amino acid identity, and the differences mainly reside in the C terminus near the transmembrane region. Both of SORDD1 and SORDD2 are predicted to have a typical C3HC4 RING domain and position C165 is predicted to be the active center. (b)RNA sequencing and qPCR results of w1118 flies show the expression of sordd1 and sordd2 in different tissues. (c) Fractionation assay to show SORDD1 is a membrane protein. A Myc tag was fused to the N terminus of SORDD1 and S2 cells were transiently transfected with Myc-tagged SORDD1.S, supernatant. P, pellet. (d) Confocal images show the cellular localization of SORDD1.S2 cells were transiently transfected with Myc-tagged SORDD1 (red), and labeled with ER marker Calnexin (green) and DAPI (blue). Scale bar, 10 μm. (e) SORDD1/2 also suppresses retinal degeneration induced by earlier expression of wild-type Rh1 and Rh1P37H. Light photomicrographs show adult eye morphology of control (GMR-Gal4/+), and flies overexpressing wild-type Rh1 (GMR-Gal4/UAS-Rh1) or Rh1P37H (GMR-Gal4/UAS-Rh1P37H), together with SORDD1 (GMR-Gal4/UAS-Rh1;UAS-sordd1/+ and GMR-Gal4/UAS-Rh1P37H;UAS-sordd1/+) or SORDD2 (GMR-Gal4/UAS-Rh1;UAS-sordd2/+ and GMR-Gal4/UAS-Rh1P37H;UAS-sordd2/+). Scale bar, 100 μm. (TIF) [file pgen.1009172.s002.tif]

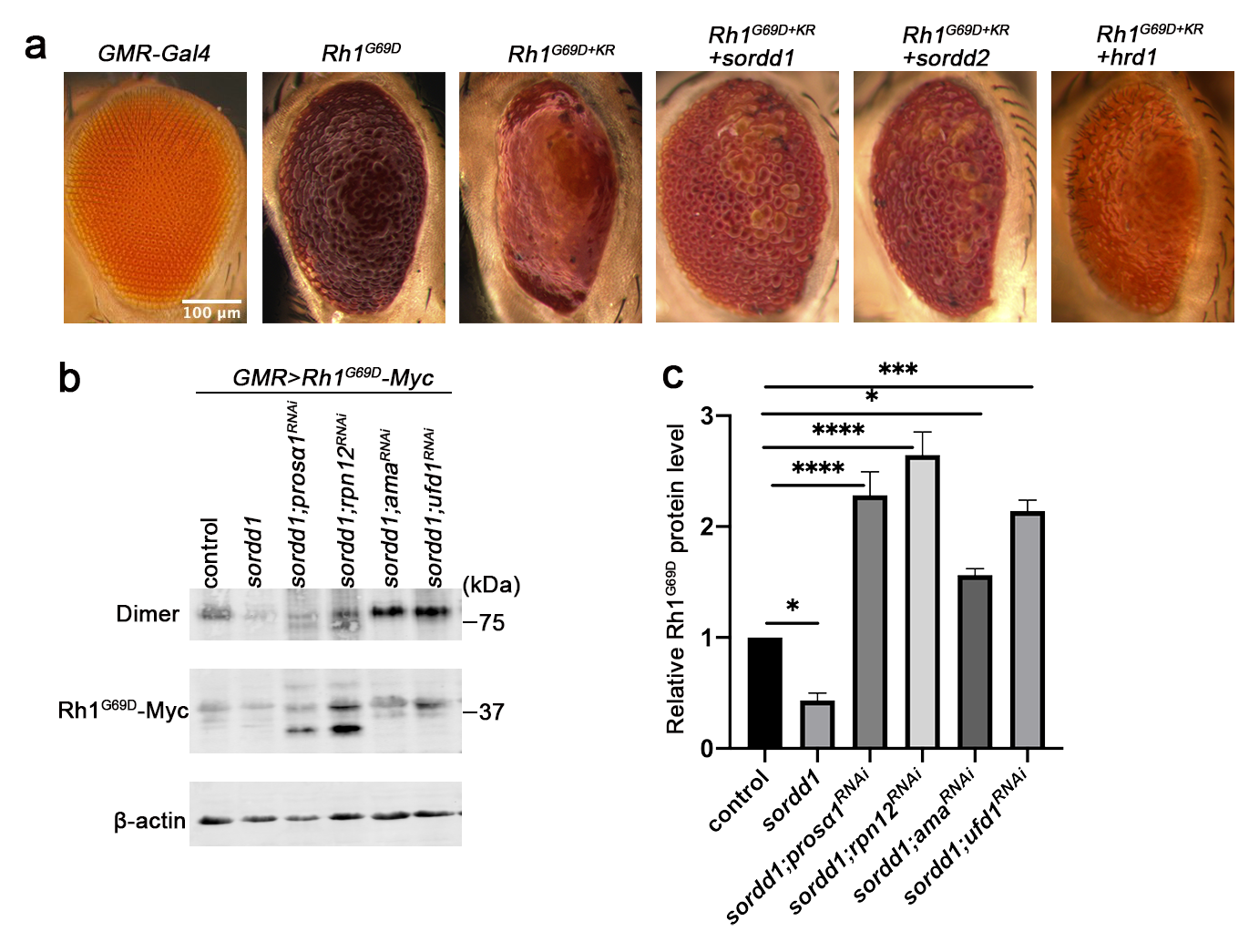

Supplement: S3 Fig — Potential ubiquitination sites of Rh1G69D are mutated by substitution of all 15 cytosol lysines and 2 luminal lysines with arginines (Rh1G69D+KR). (a) the adult eye morphology of wild type (GMR-gal4/+) and flies overexpressing Rh1G69D (GMR-Gal4/UAS-Rh1G69D), Rh1G69D+KR (GMR-Gal4/UAS-Rh1G69D+KR) together with sordd1 (GMR-Gal4/UAS-Rh1G69D+KR;UAS-sordd1/+), sordd2 (GMR-Gal4/UAS-Rh1G69D+KR;UAS-sordd2/+) or hrd1 (GMR-Gal4/UAS-Rh1G69D+KR;UAS-hrd1/+). Scale bar, 100 μm. (b) Blocking VCP and proteasome components lead to the accumulation of Rh1G69D when SORDD1 are expressed. Western blot analysis of head extracts from 1-day-old flies shows the levels of Rh1G69D–Myc in GMR>Rh1G69D-Myc sordd1 (GMR-Gal4 UAS-Rh1G69D-Myc UAS-sordd1/+) flies increase by knocking down proteasome subunits Prosα1 (prosα1RNAi, GMR-Gal4 UAS-Rh1G69D-Myc UAS-sordd1/+;UAS-prosα1RNAi/+) or Rpn12 (rpn12RNAi, GMR-Gal4 UAS-Rh1G69D-Myc UAS-sordd1/+;UAS-rpn12RNAi/+), component of VCP complex UFD1 (ufd1RNAi, GMR-Gal4 UAS-Rh1G69D-Myc UAS-sordd1/+;UAS-ufd1RNAi/+) or Amalgam (Ama), one of the genes identified in the RNAi screen (amaRNAi, GMR-Gal4 UAS-Rh1G69D-Myc UAS-sordd1/+;UAS-amaRNAi/+). Note Rh1G69D can form dimer. (c) Statistic results of b, Error bars indicate SEM; n = 3, *p<0.1, ***p<0.001, ****p<0.0001 (one-way ANOVA, Sidak's multiple comparisons test). (TIF) [file pgen.1009172.s003.tif]

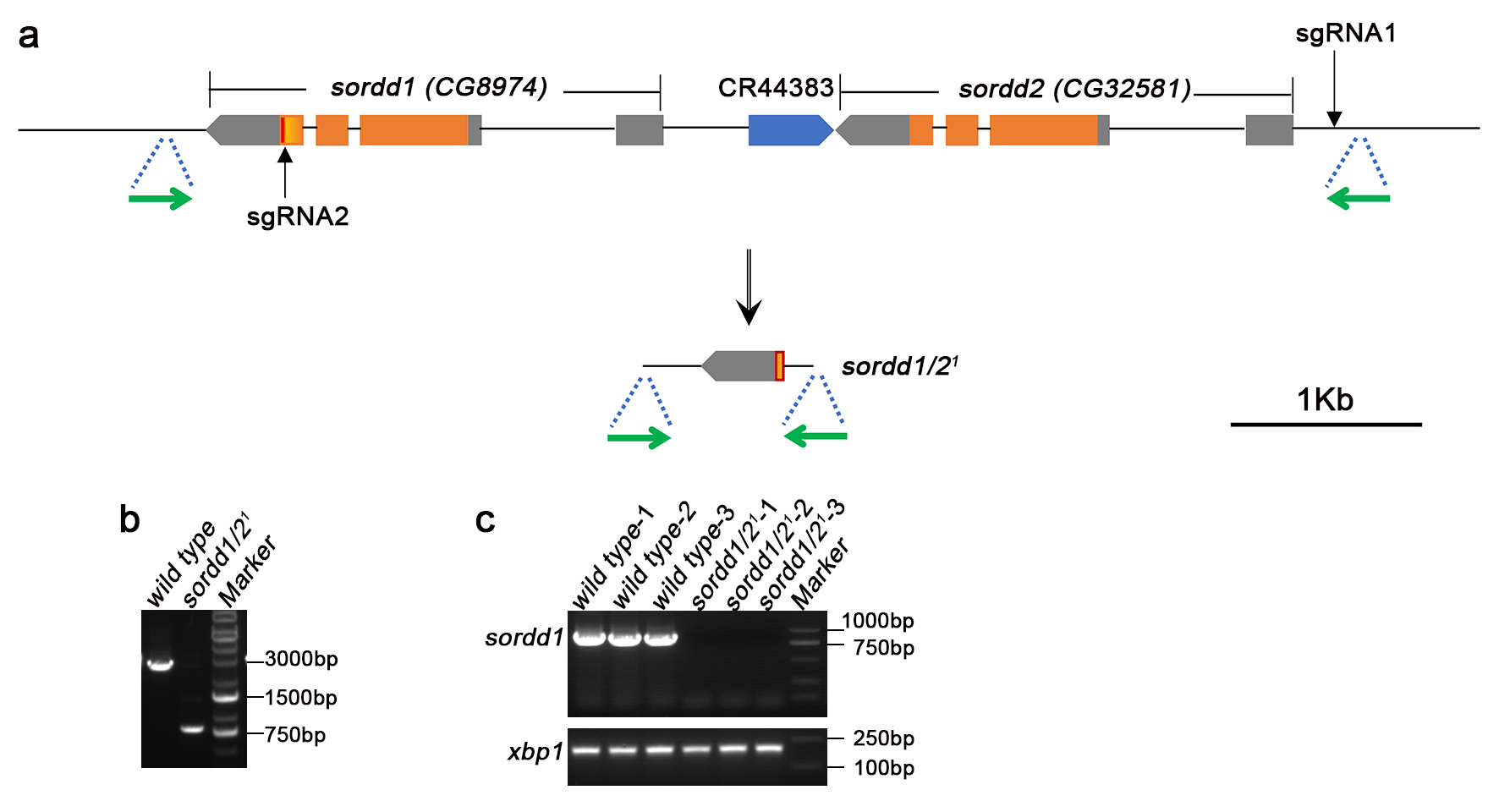

Supplement: S4 Fig — (a) Scheme for generating the sordd1/2 deletion by CRISPR-Cas9 system, the two sgRNA recognition sites and positions of the DNA primers used for PCR (arrows) are indicated. (b) PCR products of ~800 bp were obtained from sordd1/21 mutants with successful deletion of sordd1 and sordd2 loci. (c) The verification of sordd1/21 by RT-PCR.RT-PCR products of 834 bp were obtained from full-length sordd1 cDNA in wild type while absent in sordd1/21. (TIF) [file pgen.1009172.s004.tif]
