# Supplementary material for: Suppression of retinal degeneration by two novel ERAD ubiquitin E3 ligases SORDD1/2 in Drosophila
Source: PLoS Genet. 2020 Nov 2;16(11):e1009172. doi: 10.1371/journal.pgen.1009172 (PMC7660902; doi:10.1371/journal.pgen.1009172)
Supplement: Attachment — Submitted filename: Response to reviewers 2020 Xu et al.docx [file pgen.1009172.s017.docx]

Reviewer #1: In their article Wang and colleagues investigate the role of 2 ER resident E3 ubiquitin ligase SORDD1 and SORDD2 in retinal degeneration induced by Rh1G69D mutation, a model of adRP. By using a gain of function screen in animal expressing Rh1G69D, they have identified SORDD1/2 as suppressor of retinal degeneration. They further show that overexpression SORDD1/2 reduce ER stress and promote the degradation of Rh1G69D. Genome wide RNAi revealed that SORDD1 function requires VCP and the proteasome but not Hrd1 complex. Finally, crispr generated deletion of SORDD1/2 show no phenotype on its own but combined with hrd1 mutant exhibited enhanced degeneration and photoreceptor dysfunction in Rh1G69D mutant. This suggests that SORDD1/2 work in parallel with Hrd1 to degrade Rh1G69D.
The paper is interesting and the data are novel. However, some of the claims are not sufficiently supported by the data. In several cases, I was not fully convinced by the data provided and several data are not quantified.

Major comments
In figure 1, the authors state that Rh1G69D is overexpressed weakly by GMR promoter. I am surprised by this statement because GMR is a strong pan retinal driver expressed not only in photoreceptors but in all retinal cells. The authors should support their claim by showing expression level.
From multiple transgenic lines, we chose an *UAS-Rh1^G69D^* insertion that results in a weaker “glossy eye” phenotype for genetic screening. We assume that this weaker phenotype results from lower levels of Rh1^G69D^ protein, due to the insertion site of UAS transgene. However, because we did not specifically measure Rh1^G69D^ protein levels, we revised this sentence to “Rh1^G69D^ was ectopically overexpressed using *GMR-Gal4* system” (Page 6, lines 89-90). We apologize for the unintentionally misleading statement.

In figure 1b-k, the level of protection is not quantified here. Eye size and photoreceptor number should be quantified. The authors should also include a control with an overexpression control line such as UAS-GFP.
Figure1b-k indicates whether a factor can suppress the glossy eye phenotype caused by Rh1^G69D^, which can be clearly visualized by light microscopy. Moreover, the phenotype is representative and almost completely penetrance for this phenotype. Despite difficulties in quantifying the glossy eye phenotype, as suggested, we accomplished this using scanning electron microscopy (SEM) to show that the morphology of the compound eyes were consistent with light microscopy. We then counted the number of ommatidia in the middle 3 rows to indicate the level of protection. Results obtained via this SEM analysis are the same as the representative images. We have included these results in Figure S1 and highlighted text (Page 6, lines 91-92,). In addition, two control images, namely *GMR>GFP* (*GMR-gal4/+;UAS-GFP/+*) (1c) and *GMR>Rh1^G69D^+GFP* (*GMR-Gal4 UAS-Rh-1G69D/+;UAS-GFP/+*) (1h), are now included in Fig. 1.

What is the contribution of retinal pigment cell versus photoreceptor in this phenotype.
We used Rh1 as a marker of photoreceptor cells and RdhB as a marker of pigment cells, and assessed levels of these two proteins in adult wild-type and *GMR>Rh1^G69D^* flies by western blot assay. We found decreased levels of both proteins in *GMR>Rh1^G69D^* flies compared to controls. We think that both photoreceptor cells and pigment cells are damaged in *GMR>Rh1^G69D^* models. However, the contribution of different cell types is not very critical to this model, as endogenous Rh1^G69D^ expression is restricted to photoreceptor cells. Because of this, we did not include these data in the manuscript.
Interestingly SORDD1 is expressed ubiquitously but its role seems restricted to photoreceptor cells. Could SORDD1 play a role in other cell types or other misfolded proteins?
Because we used the *GMR>Rh1^G69D^* model and the gain of function screen, we only characterized SORDD1 function in photoreceptors. Actually we extracted RNA from different tissues of wild-type flies and found that *SORDD1* is ubiquitously expressed (Fig. S2b). So, we believe that SORDD1 may play a role in quality control for a variety of proteins in multiple cell types. Moreover, a recent report (PMID: 32738194) has shown that RNF185, the homologue of SORDD1, is involved in quality control for a subset of membrane proteins. We further discuss this point in the discussion section (Page 12, lines 271-273).

At this stage, there is not enough evidence to claim that SORDD1 act in the ERAD complex. All is based on the localization at the ER with calreticulin. However, the images provided does not allow to see if there is any cytoplasmic localization of SORDD1/2. These ubiquitin E3 ligases could simply be required for ubiquitination and proteasome function in the cytoplasm. What is the global protein ubiquitination profile on SORDD1/2 mutants?
We did not detect physical binding of SORDD1 to any known ERAD components, including the HRD1 complex, Herp, Der1, and the VCP complex, thus we do not claim that SORDD1 functions in an ERAD complex. Our data suggest that SORDD1 functions in degradation of misfolded Rh1, which requires both VCP complex and proteasome activities. We provided new, higher-quality images to show that SORDD1 does completely co-localize with Calnexin in the ER (Fig. S2d). Moreover, we performed a fractionation assay and showed that SORDD1 is a membrane protein associated with the fraction containing Calnexin (Figure S2c). We generated a truncated version of SORDD1/2 that lacks the C terminus transmembrane domain and found that SORDD△TM could no longer rescue GMR>Rh1^G69D^. These data indicate that ER localization is essential for SORDD1’s function.

SORDD1 may play a role in quality control for a variety of endogenous membrane proteins in multiple cell types, as is the case for its mammalian homologue, RNF185. Although it is important to identify other endogenous substrates for SORRD1, we think this is out of the scope of this manuscript, and therefore we did not examine global protein ubiquitination levels in SORDD1/2 mutants. Instead, we cite a recent report (PMID: 32738194) showing that RNF185 is involved in quality control of the membrane proteins CYP51A1 and TMUB2, and discuss this point in the discussion section (Page 12, lines 271-273).

Figure 2a should be quantified.
In the revised manuscript we now quantify levels of Rh1^G69D^-Myc, Xbp1-GFP, and ATF4-mCherry fluorescence intensity (Fig. 2b). We also added data from CG8141 as a control (Figs. 2a and 2b). These results are shown in the new Figure 2a-b and corresponding text (Page 7, lines 130-131).

Figure 3b, c. Photoreceptor degeneration can sometimes undergo below an intact cornea so the authors should show photoreceptor count for these conditions.
As suggested, we cut TEM sections including *GMR>Rh1^G69D^, GMR>Rh1^G69D^ sordd1, GMR>Rh1^G69D^ sordd1+hrd1^RNAi^, GMR>Rh1^G69D^ sordd1+hrd3^RNAi^, GMR>Rh1^G69D^ srodd1+hrd1^1^/hrd1^1^, and GMR>Rh1^G69D^ sordd1+hrd1^1^/hrd1^1^* to show the morphology of the photoreceptor cells, and confirmed that photoreceptor degeneration is rescued by expression of SORDD1 independent of HRD1/HRD3. We added these results to Figure 3c, and quantified the number of rhabdomeres in Figure 3d. For Fig. 3b, because of the dramatic cell death seen in flies expressing *Rh1^G69D^* and *srodd1* with ter94 and rpn12 RNAi (as with *GMR>Rh1^G69D^* flies), we do not think it is necessary to conduct the TEM experiments. These results are shown in Fig 3c and 3d with corresponding text (Page 22, lines 631-633).

Figure 4c. Deep pseudopupil can be difficult to distinguish in particular for quantification. Please show raw images or use an alternative for quantification such as cornea neutralization technique.
The deep pseudopupil analysis is widely used in *Drosophila* retinal degeneration studies and quantification of deep pseudopupil is often used to indicate levels of degeneration (PMID: 19805114, PMID: 29425495, PMID: 30265717). However, it is true that the deep pseudopupil may only reflect the compactness of the rhabdomere, so we cut TEM sections on Day 20 and confirmed that our deep pseudopupil analysis is correct. This evidence, in combination with quantification of DPP and TEM images, is enough to conclude that double mutations of *sordd1/2* and *hrd1* caused retinal degeneration. Since we used white eye flies, the cornea neutralization technique cannot be used. We showed representative deep pseudopupil images of wild-type and *sordd1/2^1^;hrd1^1^* flies at Day 15 as a reference.

Minor comments
In first paragraph the authors use "glassy eye phenotype" I think we use glossy instead.

We have changed this to “glossy eye phenotype” as suggested (Page 6, lines 91).

Reviewer #2: The submitted work by Xu et al. proclaimed the identification of novel E3 ligases, SORDD1/2, facilitating Rhodopsin 1 (Rh1) homeostasis in a Drosophila model and prevent mutant Rh1 induced retinal degeneration. Several rh1 mutations that fail to be correctly folded and transported to the apical membrane of photoreceptor cells could accumulate in the ER and trigger ER stress. The authors provide some evidence to argue that SORDD1/2 function in parallel with the HRD1 E3 ligase, whose action and other cofactor help mark the ubiquitin chains and provide a channel to allow the retrotranslocation of that misfolded rhodopsin to the cytoplasm. While this study's finding might be potentially significant, several issues need to be resolved before publication.

Major points:
1. The most puzzling question in the paper is that their finding contradicts to a prior report by Gricius et al. (2010) PLoS Genet. 6(8):e1001075. In that paper, authors used Rh1P37H to model a common ADRP mutation that mimicking a typical class II rod opsin mutation P23H, which is misfolded in the ER and processed by ERAD. However, they showed that by knocking down VCP/TER94, or by pharmacological treatment of either VCP or proteasome inhibitors, the retinal degeneration caused by Rh1P37H was suppressed. Although this study is using a different ADRP allele, the effect of VCP/TER94 is the opposite. Given that both alleles showed ER stress, the discrepancy shall be resolved experimentally. The authors should test Rh1P37H allele to demonstrate that SORDD1/2 are indeed genuine E3 ligases that function to process Rh1. Importantly, the authors shall bring this issue in the discussion. Furthermore, the only result that links TER94 function in SORDD1-mediated Rh1 degradation is a single RNAi allele analysis in Figure 3b, which is insufficient. TER94 has been shown to interact with several ER membrane components, the authors shall evaluate additional TER94 alleles and test if TER94 could physically interact with SORDD1.

We are aware of the paper (Gricius et al. (2010) PLoS Genet. 6(8):e1001075.). In that study the authors also found that VCP is required *in vivo* for clearance of misfolded RH1^P37H^. This is consistent with our conclusion that VCP plays a key role in ERAD-mediated clearance of Rh1. However, they showed that by knocking down VCP/TER94, or by pharmacological treatment of either VCP or proteasome inhibitors, the retinal degeneration caused by Rh1^P37H^ was suppressed. To explain this opposite observation, we first asked whether SORDD1 could suppress RH1^P37H^-mediated retinal cell death. We found that both SORDD1 and SORDD2 could suppress the eye damage caused by RH1^P37H^ or by Rh1^G69D^ (Fig. S2e), and reduced levels of RH1^P37H^-Myc. Therefore, we experimentally ruled out the possibility that misfolded RH1^P37H^ and Rh1^G69D^ are different substrates of ERAD and/or different mechanisms of associated cell death. The new results are included in Figure S2e and associated text (Page 7, lines 116-118).

The Rh1^P23H^ mutation, representing a prototypical model for studying proteotoxicity, has been extensively studied in rodents. In our case, boosting E3 ligase activity (including SORDD1 and HRD1) accelerated the clearance of misfolded Rh1, which is consistent with the *Rho^P23H^* mice models. Unlike the fly RH1^P37H^, proteasome levels and activity were reduced in the retinas of *Rho^P23H^* mice, and induction of proteasome activity via the genetic deletion of *Atg5* or a selective phosphodiesterase-4 inhibitor (rolipram) alleviates retinal cell death (PMID: 16901789 and 31320609). These conflicting results might be due to regulation of general cellular proteostasis by the proteasome and multiple function of VCP. Therefore, inhibition of VCP or the proteasome could suppress a cell death pathway downstream of misfolded rhodopsin. For example, VCP is required for autophagy (PMID: 20008565), and disruption of VCP blocks autophagic flux. This suppresses Rho^P23H^-induced retinal degeneration (PMID: 16901789). These data could explain the different observations between our study and the Ueffing group. However, in flies with the accelerated clearance of misfolded Rh1 by overexpression of SORDD1, disruption of VCP or the proteasome dramatically blocked Rh1 degradation, thus reducing the eye damage caused by Rh1^G69D^. We further discuss the potential roles of VCP and the proteasome in RH1^P37H^/Rho^P23H^-induced cell death in the discussion section (Page 12, lines 249-256).

To further support our ter94 data, we tested another ter94 RNAi line (VDRC) and obtained the same result. These data are presented in the new Figure 3b.

We also performed mass spectrometry with GFP-SORDD1 to find factors that associated with SORDD1, but did not identify TER94. Furthermore, and as suggested by reviewers, we tested for interaction between TER94 and SORDD1 by expressing SORDD1 with a Myc tag and TER94 with a Flag tag in S2 cells, and checked for interaction by co-IP. We did not find a physical interaction (see figure below). It is reasonable to conclude that the VCP complex does not need to physically interact with the E3 ligase in the ERAD pathway, but it instead functions downstream of the E3 ligase.

2. Another concern is the data used to prove that SORDD1 is an ER membrane protein (Figure S1b). As this study claims, SORDD1 is an ER membrane component for the first time, and there is no prior report of this E3 ligase, robust analyses are required for scrutinizing. The image of the S2 cell shown in S1b has some caveats. First, the manuscript didn’t mention how eGFP was tagged to SORDD1. Second, the authors didn’t prove that the localization of SORDD1-GFP fusion protein remains the same as the endogenous SORDD1. Third, the GFP signals from the fusion protein and anti-Calnexin staining were exceedingly saturated. It is risky to use that overlapping to suggest SORRD1 is an ER membrane component. To convince readers that this E3 ligase indeed resides in the ER membrane, an antibody that could recognize the endogenous SORDD1 is preferable. Alternatively, if the antibody is not available or not work, it is necessary to provide either co-IP or proximity-based labeling results with a smaller tag to demonstrate that SORDD1 is indeed associated with a prominent ER protein.

As we were unable to generate high-affinity SORDD1 antibodies, we fused a Myc tag to the N-terminus of SORDD1 and expressed this tagged protein in S2 cells. We provide new, higher-quality images to show that SORDD1 does completely co-localize with Calnexin in the ER (Fig. S2d). Moreover, we performed a fractionation assay and biochemically showed that SORDD1 is a membrane protein that associates with the fraction containing Calnexin (Figure S2c). These data demonstrated that SORDD1 is a prominent ER protein.

3. The manuscript indicated that overexpression of E3 ligase CG8141 did not suppress Rh1G69D-induced eye phenotype. As both CG8141 and sordd1 are orthologous to RNF185 and RNF5, it will be more convincing to show that CG8141 has functionally deviated from sordd1/2 on the issue of Rh1 processing. The ERAD reporters used in Figure 2a should include GMR>Rh1G69D+ CG8141.

We added the CG8141 data to the ER stress reporters in the new Figure 2a, and added a quantification to Figure 2b as suggested.

4. The manuscript underscores SORDD1/2 E3 ligase activity involves in Rh1 homeostasis. Interestingly, Figure S2a showed that SORDD1 expression still partially suppresses Rh1G69D-KR-induced rough eye phenotype. As the authors noted that Rh1G69D-KR is ubiquitin-resistant, several issues need to be clarified for this data. First, the authors didn’t mention what lysine residues of this transgenic fly have been changed. The detail of generating this ubiquitin-resistant allele should be included. Second, an IP experiment with anti-ubiquitin detection is essential to demonstrate that this KR allele is free from ubiquitination. Third, because the authors indicated that Rh1G69D-KR is ubiquitin-resistant, the authors should explain how does SORDD1 remains capable of the partial rescue?

We mutated potential ubiquitination sites in Rh1^G69D^ by substituting all 15 cytosolic lysines and 2 luminal lysines with arginines. We then assessed levels of Rh1^G69D+KR^ ubiquitination by IP with Myc-tag followed by anti-ubiquitin detection. To our surprise, compared to Rh1^G69D^, the level of Rh1^G69D+KR^ ubiquitination was very low, but not completely gone (see figure below). This version of Rh1^G69D^ has 3 lysines within the transmembrane domain that were not mutated to arginine, and it is possible that these lysines could be ubiquitinated by SORDD1. This may explain why SORDD1 remains capable of the partial rescue.

5. The authors should experimentally clarify whether the modulation of sordd1 would affect endogenous hrd1 expression or vice versa, given that these two E3 ligases serve in a complementary manner.

We did perform qPCR experiments and found that *hrd1* mRNA levels did not change when sordd1/2 was knocked out. This indicates that the transcription levels of *sordd1/2* and *hrd1* are not correlated. We did not include these negative data in the revised manuscript.

6. There are several issues in those compound eye micrographs. First, I am troubled by the scale bar throughout the manuscript. If the scale bar is correct, the A-P dimension of a fruit fly eye is greater than 3 mm, which is impossible. Second, the micrographs of the same genotype often showed inconsistent morphology. For example, while both Figure 1d and Figure 2d showed the external eye morphology of GMR>Rh1G69D+hrd1, one can tell that in Figure 1d showed mild roughness, but the panel showed the same genotype in Figure 2d looked normal. Another example has to do with GMR>Rh1G69D+sorrd1 (Figure 1e) or sorrd2 (Figure 1f), which showed normal external eye morphology. However, when looking at the same genotype from the corresponded panels in Figure 2d, the rough eye phenotype was evident. Such irregularity of phenotype from the same fly strain raises great concerns, especially for interpreting the issue regarding hrd1 and sorrd1 function in parallel to processing Rh1, as shown in Figure 3c. The authors shall adopt methods with either a better resolution or with a quantifiable measurement to improve the quality.

We apologize for mistakenly including inaccurate scale bars in the compound eye micrographs due to a wrong setting of our light microscope. We thank the reviewer for pointing this out, and have adjusted the scale bar in the new manuscript.

In figure 1d-f, *UAS-hrd1*, *UAS-sordd1,* and *UAS-sordd2* are second chromosomal insertions, whereas in figure 2d, we used a single transgene on the third chromosome. Although both transgenes showed dramatic suppressing effects, the suppression efficiencies are slightly different. In the revised manuscript, we provide higher-quality images using the same *UAS-hrd1*, *UAS-sordd1,* and *UAS-sordd2* transgenes (third chromosomal insertion) for both Figure 1 and Figure 2. We also used scanning electron microscopy to show the morphology of the compound eyes, and quantified the number of ommatidia in the middle 3 rows to indicate the level of protection (see new Figure S1). Furthermore, in Figure 3c we provide TEM images of the corresponding lines and quantified the number of rhabdomere in each ommatidia.

7. The IP data in Figure 2e suggests the expression of SORRD1C165S could lower the ubiquitination level of Rh1G69D-Myc. However, it is apparent that anti-Myc IP result also showed much lower Rh1G69D-Myc protein levels. Because the experiment was performed in rpn12 knockdown background, the authors were awarded this problem, but chosen this data to argue the E3 ligase activity of SORRD1 is crucial for Rh1 processing does raise some concerns. The authors should provide data with a comparable amount of Rh1G69D-Myc in wild type SORRD1 and SORRD1C165S background to substantiate their findings.

The severity of eye damage is different when you compare *Rh1^G69D^-Myc/rpn12^RNAi^* or *Rh1^G69D^-Myc, SORDD1^C165S^/rpn12^RNAi^* to *Rh1^G69D^-Myc, SORDD1^C165S^/rpn12^RNAi^* flies. Because of this, we used the same number of fly eyes as input in the last manuscript, which does not guarantee the same level of Rh1^G69D^-Myc loading. We repeated this ubiquitination assay several times, and adjusted the loading of each sample according to Rh1-Myc levels. We could readily detect elevated levels of Rh1^G69D^–Myc ubiquitination when wild-type SORDD1 was expressed. These results, and their quantification, are shown in Figure 2f and 2g.

8. One highlighted point in the manuscript is that SORDD1/2 and Hrd1 function in parallel and complementary to maintain Rh1 homeostasis. While the authors showed that loss of all three genes leads to age-dependent photoreceptor cell degeneration (Figure 4d), it is not clear whether this is due to the disturbance of Rh1 homeostasis. Furthermore, the main text or the figure legend didn't indicate whether these panels in Figure 4d are from wild type or Rh1G69D backgrounds. If this is from the wild type background, the authors should compare the endogenous Rh1 protein levels right before the onset of photoreceptor degeneration to address this issue.

To emphasize that the panels in Figure 4c-d are images from a wild-type background, we added “we examined photoreceptor cell integrity in *sordd1/2^1^* and *hrd1^1^* mutants that expressed wild-type rhodopsin.” (Page 12, lines 239-240). We also compared levels of endogenous Rh1 protein in wild type, *sordd1/2^1^*, *hrd1^1^*, and *sordd1/2^1^;hrd1^1^* flies 3 days after eclosion. In *sordd1/2^1^;hrd1^1^* mutants, Rh1 levels were significantly lower, whereas other phototransduction-related proteins, such as TRP and INAD, did not change (see new Figure 4e and 4f). This indicates that the disturbance of Rh1 homeostasis occurred before retinal degeneration and that disruption of Rh1 homeostasis might lead to the age-dependent photoreceptor cell degeneration in *sordd1/2^1^;hrd1^1^* mutants. We added these data to Figure 4e and 4f, and in the main text (Page 9-10, lines 192-199).

9. Figure 1a is incorrect because the EP lines of SORDD1/2 are in the X chromosome. This kind of error shall avoid.

We apologize for this misleading figure. While the fly ORFs are all third chromosome insertions, the EP lines are inserted on the X, 2^nd^ and 3^rd^ chromosomes. It is difficult to include each chromosomal insertion in one figure, so we chose third chromosomal insertion lines as representative data. To avoid this misunderstanding, we added “The representative strategy of screening for UAS-cDNAs inserted on the 3^rd^ chromosome is shown.” to the legend of Figure 1a (Page 21, lines 581-582).

Minor points:
1. In the first Result section, the authors stated – "Rh1G69D was weakly expressed in undifferentiated photoreceptor neurons (GMR>Rh1G69D)"– is misleading. First of all, GMR-Gal4 is considered a strong driver, and there is no quantitative index or means to make such a statement. Second, GMR-Gal4 drives UAS transgene expression in all cells of differentiating ommatidia posterior to the morphogenetic furrow, not limiting to photoreceptor neurons.

We change this sentence to “Rh1^G69D^ was ectopically overexpressed using the GMR-Gal4 system” to avoid this misleading statement (Page 6, lines 89-90).

2. The molecular weight of Rh1G69D in Figure S2b and Figure 2b seems quite a different judging from the marked MWs. A major band (blow 37 kD mark) could be found in the third and the fourth lanes of S2b. Is that an unspecific band or a processed Rh1?

We used pupa eyes to perform the western blot in Figure 2c (previous 2b), whereas adult heads were used in Figure S3b (previous S2b). This difference has been clarified in the figure legends. The different band sizes of Rh1 may reflect different modifications of Rh1 in photoreceptor cells at different developmental stages. We think that major band is a processed Rh1 band because this band only appears when the proteasome component is suppressed such as rpn12 knock down or prosalpha1 knock down.

3. Figure 2e and 2f appeared in the text without mention anything about rpn12, which only seemed later. The writing flow should be fixed.

We used *rpn12^RNAi^* to inhibit proteasome activity and then examine ubiquitination levels before showing the suppressor screening results, where the definition of RPN12 appearing. We now added “The proteasomal degradation of Rh1^G69D^ was inhibited by knocking down the proteasome component Rpn12 (*rpn12^RNAi^*).” to the figure legend of Figure 2f (when it first appears) (Page 22, lines 611-612).

4. Toward the end of the first paragraph in the third section of the Result, it should be “when knocked down alone” instead of “when expressed alone” because the corresponded experiments were using the RNAi approach.

We have changed it to “when knocked down alone” (Page 8, lines 157).

5. The authors should provide a citation of experimental data to demonstrate that rpn12 knockdown would affect proteasome function.

In the paper entitled “Actin Cytoskeletal Organization in Drosophila Germline Ring Canals Depends on Kelch Function in a Cullin-RING E3 Ligase” (PMID: 26384358), knocking down *rpn12* was shown to inhibit proteasome activity. We cite this reference in the Figure 2f legend (Page 22, lines 612).

6. The authors shall avoid using “cell death” in their text because none of the provided data includes any cell death assay. An example could be found to the end of section 3 in the Result.

We change “cell death” to “cell degeneration” or “retinal degeneration” in the revised manuscript.

7. The genotypes in Figure 3b and 3c are confusing. While the labeling of sordd1 was no difference in all panels, the genotypes stated in the Figure legend were either UAS-sordd1 or simply sordd1 (is this indicating the EP line?). The authors need to make sure the genotype is correctly described.

All *sordd1* alleles in Figure 3 are a combination of *GMR-Gal4* and *UAS-sordd1*. We omitted “UAS” in 3 places in the figure legend. This has been fixed in the revised figure legend.

Reviewer #3: The manuscript by Jaiwei Xu, Haifang Zhao, and Tao Wang used an overexpression screen to identify two redundant ubiquitin ligases SORDD1/2 as additional factors that contribute to the degradation of misfolded opsins. By-and-large the experiments are well controlled and the data convincing. Identification of this novel HRD1/3 independent ER degradation pathway is intriguing and an important. I have just a few comments that should be straightforward to address.


1. For the ERG analysis (fig. 5) the flies were maintained under constant light.
It seems important to note that constant light treatment triggers elevated ER stress (PMID: 30015618). Is that elevated ER stress necessary to achieve the strong ERG phenotype reported? Was the same pre-treatment used for the deep pseudo pupil analysis?
It has been reported that retinal degeneration is slow in the endogenous adRP model *ninaE^G69D^/+* under a normal light-dark cycle (~6 weeks, PMID: 7708777), whereas constant light accelerated retinal degeneration (~3 weeks, PMID: 19805114). For these experiments we used constant light to accelerate retinal degeneration in *ninaE^G69D^/+* flies. Although we are not sure if this is due to the elevated ER stress caused by constant light treatment, wild-type controls (red eyes) that were raised under the same constant light condition (white light of ~700 Lux at 25ºC) do not exhibit severe retinal degeneration, even after 30 days. These rearing conditions are described in the methods section.

These are the only experiments for which we used constant light. In other places, such as the deep pseudopupil analysis, flies were maintained under normal light-dark cycle condition. We added “All flies were maintained under 12 h light /12 h dark cycles at 25ºC unless mentioned.” in the first methods section (Page 13, lines 288).

2. The title is a bit misleading, as it is only the overexpression of SORDD1 that results in suppression of the retinal degeneration phenotype. Related to this; it may be old-fashioned, but the name SORDD1/2 seems a bit unfortunate. Classically, fly genes have been named for their loss-of-function rather than their overexpression phenotypes.
It is true that fly genes are typically named for their loss-of-function rather than their overexpression phenotypes. This is because genetic screens have historically been loss-of-function screens.
We have used a gain-of-function screen, and feel that if an allele is obtained via this methodology, it is acceptable to name the gene after its gain-of-function phenotype. There are examples of this. A sleep-promoting molecule encoded by a gene they named nemuri (Japanese for “sleep,” abbreviated nur) was identified using an overexpression screen (PMID: 30705188). We now avoid this misleading name by change the full name of SORDD1 to Suppression Of Retinal Degeneration Disease 1 upon overexpression, as highlighted in the results section. (Page 6, lines 99)

3. I do not understand why HRD1-independence of SORDD1 activity disproves that "that HRD1 may work as the retro-translocation channel for this kind of multi-span membrane substrate". author statement). It just seems to prove that it is not the only one especially given that the endogenous HRD1 and SORDD1 genes appear to be part of two redundant pathways for Rho[G69D] degradation (Figure 4)
The retro-translocation channel is the largest unsolved question in the ERAD field, and recently many studies have claimed that HRD1 may function as both an E3 ligase and a retro-translocation channel for ERAD-L substrates at the same time (PMID: 27321670, PMID: 32094691, PMID: 32327568). Our results indicated that for the ERAD-M substrate, Rh1^G69D^, HRD1 did not serve as the core retro-translocation channel for the SORDD1 pathway (if a retro-translocation channel is required for SORDD1-associated substrate degradation). To avoid this misunderstanding, we deleted the relevant sentence and only stated that “SORDD1 suppressed Rh1^G69D^-induced ER stress and cell damage independent of HRD1 and HRD3.”.

4. There is a significant literature on the mammalian RNF5 / RNF185 homologs of SORDD1/2, for example in the context of cystic fibrosis or immunity. Given the functional conservation shown by the authors, known functions of these mammalian homologs should be discussed and compared to the current findings.

We further discuss the function of SORDD1 homologues, RNF5 and RNF185, in the discussion section. We also cite and discuss a new paper (PMID: 32738194) about RNF185 and quality control of endogenous ER membrane proteins. We added these results to Figure 4e and 4f, and the main text (Page 12, lines 264-273).

5. Regarding the Western blots shown in Figure 2e: Elevated ubiquitination of Rh1G69D–Myc in the SORDD1 Wt compared to SORDD1-C165S expressing flies is clear, but how do the authors explain that Rh1G69D–Myc is not accumulating to the same level given that proteasomal degradation is inhibited. This should be addressed.
In Figure 2e (Figure 2f in revision), as we are comparing the ubiquitination levels of Rh1^G69D^–Myc, we performed the ubiquitination assay by adjusting the amount of sample loaded based on the level of Rh1^G69D^–Myc, not the amount of total protein. Therefore, in Figure 2, accumulation of Rh1^G69D^–Myc is not evident, only elevated levels of Rh1^G69D^–Myc ubiquitination in SORDD1^wt^ flies. We repeated this experiment several times and added quantification of these data to Figure 2f and 2g.

Minor details:
6. The EM procedure mentions 4% paraformaldehyde and 2.5% pentanediol as fixative
Presumably the authors mean pentanedial more commonly referred to as glutaraldehyde.

We used glutaraldehyde for EM fixation. We have changed pentanediol to glutaraldehyde in the revised manuscript (Page 16, lines 375).

7. lots of typos and grammar errors e.g.:

We apologize and have reviewed the revised manuscript several times to find and correct typos and grammatical errors.

Amalgam (Ama) one of gene identified in RNAi screen (Figure legend S2)

We change it to “Amalgam (Ama), one of the genes identified in the RNAi screen” (Page 24, lines 697-698).

Presumably statistical significance was assessed rather than "applied" (Statistics section)

We change it to “statistical significance was assessed” (Page 16, lines 390).

SORDD1 function dependently on VCP and the proteasome but independently on the RD1 complex (section 3)

We change it to **“**VCP and the proteasome but not the HRD1 complex are required in SORDD1-mediated degradation of Rh1^G69D^” (Page 8, lines 146-147).

Degrading misfolded ER membrane proteins, as called ERAD-M,

We change it to “which is called ERAD-M” (Page 8, lines 161).

Mutations in hrd1 or hrd3 have no effect in eye morphology

We change it to “Mutations in hrd1 or hrd3 had no effect on eye morphology” (Page 22, lines 624).
