# Supplementary material for: Suppression of retinal degeneration by two novel ERAD ubiquitin E3 ligases SORDD1/2 in Drosophila
Source: PLoS Genet. 2020 Nov 2;16(11):e1009172. doi: 10.1371/journal.pgen.1009172 (PMC7660902; doi:10.1371/journal.pgen.1009172)
Supplement: Attachment — Submitted filename: Response to reviewers Xu et al.docx [file pgen.1009172.s018.docx]

Reviewer #2: The revised manuscript by Xu et al. has made significant improvements. However, the following issues need to be resolved before acceptance for publication.

1. The authors have responded to my earlier concern regarding the functional deviation of SORDD1/2 and another E3 ligase CG8141. The revised manuscript has included the CG8141 overexpression allele in ER stress reporters assay. As seen in Figure 2a, the signals of XBP1-EGFP and ATF4-mCherry in the bottom row (Rh1G69D-Myc + CG8141) appeared significantly enhanced and did not follow Rh1G69D-Myc's staining pattern as compared to Rh1G69D-Myc alone (2nd row from top). Because CG8141 overexpression did not worsen eye degeneration, how do the authors explain this observation? While the authors showed quantitative data in Figure 2b, given the intensity difference of the represented image in Figure 2a, and the N value was 4 in Figure 2b, the data require careful review.

The signals of XBP1-EGFP and ATF4-mCherry in Rh1^G69D^-Myc + CG8141 did not significantly enhance compared to Rh1^G69D^-Myc alone as we quantified the fluorescence intensity from multiple samples. The lumpy fluorescence signals in the bottom row of the representative picture of Fig. 2a are autofluorescence signals since no cells are in that area according to the DAPI staining. When we quantified the fluorescence intensity, we did exclude these lumpy signals. To avoid misleading the readers we choose a new representative image to replace that panel this time (see new figure 2a).

2. According to the authors’ response, Rh1G69D-KR has three lysine residues within the transmembrane domain that were not mutated to Arginine, which might explain why the expression of sordd1/2 could still partially rescue the Rh1G69D-KR-caused rough eye phenotype. This information, while no clear mechanistic insight, should be revealed in the main text.

We add the sentence “The partial rescue effect might be due to the three lysine residues remaining within the transmembrane domain of Rh1G69D+KR which could also be ubiquitylated by SORDD1/2” in the main text (Page 7, lines 140-142) to clarify this point.

Reviewer #3: The authors have appropriately addressed the majority of concerns raised by the previous round of reviews. The exception is their conclusion that SORD1/2 are ER proteins.
This is entirely based on the analysis of overexpressed SORD1/2 proteins in S2 cells. Accumulation of overexpressed proteins in the ER is not unusual. Therefore, the authors' conclusion that endogenous SORD1/2 are ER proteins is not convincingly supported by the data and the conclusion should be modified accordingly.

We could not show the staining pattern of endogenous SORDD1/2 as we were unable to generate high-affinity SORDD1 antibodies. Previous studies have revealed that RNF185-the human homolog of SORDD1/2-is an ER resident E3 ligase and functions in the ER quality control (PMID: 24019521, PMID: 32738194). Therefore, we conclude that SORDD1/2 are ER proteins not only based on our overexpression experiments but also on previous studies about its homolog and we cite these papers in our main text. Meanwhile, we deleted our conclusion that SORD1/2 are ER resident proteins as “Overexpression SORDD1/2 associate with the membrane fraction of S2 cells and colocalize with the ER protein Calnexin (Fig S2c-d). Moreover, RNF185, the human homolog of SORDD1/2, was found to be an ER resident E3 ligase and function in the degradation of transmembrane proteins in ER, suggesting that SORDD1/2 may be directly involved in degrading misfolded Rh1G69D in ER [25, 26].” (Page 7, lines 122-125).
